# Whisker row deprivation affects the flow of sensory information through rat barrel cortex

**DOI:** 10.1152/jn.00289.2016

**Published:** 2016-10-05

**Authors:** Vincent Jacob, Akinori Mitani, Taro Toyoizumi, Kevin Fox

**Affiliations:** ^1^School of Biosciences, Cardiff University, Cardiff, United Kingdom;; ^2^RIKEN Brain Science Institute, Wako, Saitama, Japan; and; ^3^Department of Neurosciences, University of California, San Diego, La Jolla, California

**Keywords:** cortical circuit, conductance, cortical column, plasticity, vibrissa

## Abstract

Whisker trimming causes substantial reorganization of neuronal response properties in barrel cortex. However, little is known about experience-dependent rerouting of sensory processing following sensory deprivation. To address this, we performed in vivo intracellular recordings from layers 2/3 (L2/3), layer 4 (L4), layer 5 regular-spiking (L5RS), and L5 intrinsically bursting (L5IB) neurons and measured their multiwhisker receptive field at the level of spiking activity, membrane potential, and synaptic conductance before and after sensory deprivation. We used Chernoff information to quantify the “sensory information” contained in the firing patterns of cells in response to spared and deprived whisker stimulation. In the control condition, information for flanking-row and same-row whiskers decreased in the order L4, L2/3, L5IB, L5RS. However, after whisker-row deprivation, spared flanking-row whisker information was reordered to L4, L5RS, L5IB, L2/3. Sensory information from the trimmed whiskers was reduced and delayed in L2/3 and L5IB neurons, whereas sensory information from spared whiskers was increased and advanced in L4 and L5RS neurons. Sensory information from spared whiskers was increased in L5IB neurons without a latency change. L5RS cells exhibited the largest changes in sensory information content through an atypical plasticity combining a significant decrease in spontaneous activity and an increase in a short-latency excitatory conductance.

**NEW & NOTEWORTHY** Sensory cortical plasticity is usually quantified by changes in evoked firing rate. In this study we quantified plasticity by changes in sensory detection performance using Chernoff information and receiver operating characteristic analysis. We found that whisker deprivation causes a change in information flow within the cortical layers and that layer 5 regular-spiking cells, despite showing only a small potentiation of short-latency input, show the greatest increase in information content for the spared input partly by decreasing their spontaneous activity.

## Abstract (precis)

Sensory cortical plasticity is usually quantified by changes in evoked firing rate. In this study we quantified plasticity by changes in sensory detection performance using Chernoff information and receiver operating characteristic analysis. We found that whisker deprivation causes a change in information flow within the cortical layers and that layer 5 regular-spiking cells, despite showing only a small potentiation of short-latency input, show the greatest increase in information content for the spared input partly by decreasing their spontaneous activity.

sensory experience dynamically reshapes cortical receptive fields. Subregions of the receptive field can be strengthened or weakened by altering the balance of sensory drive between competing inputs. In layers 2/3 (L2/3), the nature of receptive field plasticity is known to vary with the type of sensory modification set by the experimenters, but for any given pattern of deprivation, the plasticity is relatively uniform in effect across neurons (Feldman and Brecht 2005; Wallace and Fox 1999). In contrast, we recently found that a row-deprivation pattern can cause nonuniform receptive field plasticity in subtypes of layer 5 (L5) pyramidal cells (Jacob et al. 2012). The two main types of pyramidal cells in cortical layer L5 are known as thick tufted or intrinsically bursting (IB) cells and thin slender or regular-spiking (RS) cells (Chagnac-Amitai et al. 1990; Connors and Gutnick 1990; McCormick et al. 1985). In deprived columns of barrel cortex, spared whisker inputs were potentiated in IB cells together with little depression of deprived inputs, whereas RS cell responses to deprived whiskers were depressed without potentiation of spared input (Jacob et al. 2012).

What mechanisms might be responsible for the different reactions of different cell types to the same deprivation pattern? At the cellular level, these include different capacities between cells for particular synaptic plasticity processes such as long-term potentiation (LTP), long-term depression (LTD), and homeostatic plasticity; at the circuit level, these include the dynamic interaction between cells in different layers of the cortical columns, the relative strength of cortical vs. thalamic input differences, and changes in the balance between excitatory and inhibitory inputs. A previous study in mouse barrel cortex has provided evidence Hebbian and homeostatic mechanisms are recruited to different degrees in L5RS and L5IB cells (Greenhill et al. 2015). In this study we have concentrated on the differences that might arise at the circuit level.

To understand the information flow between layers, we recorded intracellularly from all layers and compared spontaneous activity and receptive fields using both conventional measures of activity (subthreshold inputs and suprathreshold outputs) and analysis of Chernoff information induced by whisker stimulation. We also analyzed response latencies to judge the relative dominance of rapid thalamic vs. slower intracortical inputs after plasticity. Finally, in a separate set of experiments, we measured synaptic conductance of evoked postsynaptic potentials (PSPs) in RS and IB cells as a first step toward understanding whether the excitatory inhibitory balance might be responsible for differing expression of potentiation and depression in IB and RS cells, respectively. Although L5RS cell responses did not potentiate above the baseline value (as reported by Jacob et al. 2012), they were specifically found to convey increased information on spared whisker stimulation due to the combination of lower spontaneous activity and potentiation of a fast excitatory component.

## METHODS

### 

#### Subjects and whisker deprivation.

Experiments were performed at Cardiff University and were approved under the UK Scientific Procedures Act 1986. Recordings were performed in 37 control and 30 deprived Long-Evans male rats. Animals were lightly anesthetized with isoflurane and had the left D-row of whiskers trimmed to length <1 mm (same length as the fur hairs) every 24 or 48 h. Whisker trimming started at postnatal *day 32–45* (P32–P45) and was continued for 10 days before recording; the trimmed whiskers were kept and glued to the whisker cut end before stimulation. Control animals were recorded at the same age as deprived animals, but the D-row of whiskers were trimmed and glued on the day of recording. For this reason, recordings from trimmed whiskers were compared with those from control D-row whiskers, whereas the control for the spared whiskers were the C- and E-row whiskers.

#### Surgery and recording procedures.

Anesthesia was induced with isoflurane and maintained with intraperitoneal injection of urethane (1.5 g/kg body wt). Anesthetic depth was monitored by reflex movements, breathing rate, and cortical activity, and if required, additional doses of urethane were injected (0.15 g/kg body wt). Body temperature was maintained at 37°C with a thermostatic heating blanket. The animal was placed in a stereotaxic frame and a 1-mm-diameter craniotomy performed over the D1-2 barrels. A separate craniotomy was made caudally away from the barrel field to insert a carbon fiber reference electrode at the cortical surface. Glass micropipettes filled with 1 M potassium acetate and 2% biocytin (50–100 MΩ) were inserted in the brain through a small opening of the dura. Recordings were performed in current-clamp mode, and the bridge was balanced manually (Axoclamp 2B). Recordings were excluded from analysis when the average membrane potential was within 50 mV of the action potential peak amplitude. Every 2.2 s between stimulation sequences, a short hyperpolarizing current (10 pA, 100 ms) was injected in the cell and the series and membrane resistance were calculated using a double exponential fit. Data from 32 L5RS cells and 38 L5IB cells were already included in a previous study with different analysis (Jacob et al. 2012).

#### Whisker stimulation.

Whiskers were deflected with the use of 9 independent computer-controlled piezoelectric actuators (Physik Instrumente, Bedford, UK) arranged in a 3 × 3 array (Jacob et al. 2012). The principal whisker and the 8 immediate neighbor whiskers were trimmed to 12-mm length and inserted 3 mm into short tubes glued onto the actuator. When the principal whisker was not at the center of the array in the control animals, the whiskers two rows or two columns apart from the principal whisker were excluded from the analysis. Each element of the stimulator has a very large range of positional adjustments due to gimbal joints, and the actuators were positioned and oriented to maintain the whiskers at their initial resting position and angle unless stimulated. Piezoelectric bender movement was controlled by a whisker stimulator driver (CED 3901) interfaced with a data acquisition interface (CED 1401; Cambridge Electronic Design, Cambridge, UK). The deflection amplitude of each actuator was calibrated with a laser displacement measurement system (Micro-Epsilon, Ortenburg, Germany). Receptive fields were mapped with sparse noise stimuli composed of pseudorandom sequences of ventral/dorsal deflections at 5 Hz (interpolated with a nonstimulation event). Five to 125 sequences (mode 50) were considered, depending on the stability of the recording. Each whisker deflection lasted 30 ms (with a 10-ms plateau phase) to avoid oscillations and was 200 μm in peak amplitude (Fig. 2*A*).

#### Cell type identification and location.

After recordings were complete, animals were perfused with fixative under deep general anesthesia. Biocytin staining of L5 cells was revealed in coronal sections (300 μm thick) using Vectastain ABC kits (Vector Laboratories, Peterborough, UK). The morphology of a subset of recorded cells was recovered and used to establish a correlation between somatic position and electrode depth from the surface of the saline solution above the pia. The border between L2/3 and L4 was found to be located at a depth of 575 μm, the border between L4 and L5 was located at a depth of 950 μm, and the border between L5 and L6 was located at a depth of 1,400 μm. The cells used for morphological characterization included part of the cells whose receptive fields were recorded plus an additional sample. We did not have a sufficient sample to classify receptive fields according to cell morphology. Instead, we used physiological criteria. A number of methods are available to classify L5 cells into IB and RS populations, but none unambiguously classify all cells. Nevertheless, the classification bias we encountered is likely to be marginal because we observed that 88% of the cells were classified into the same categories with the use of two distinct methods: these were the original method based on evoked firing (Connors and Gutnick 1982) and a more recent quantitative method based on spontaneous activity (Nowak et al. 2003). In the present study we decided to use the original method: L5 cells were identified as IB cells if they responded to current injection at least once with a characteristic burst shape, comprising high-frequency action potential decreasing in size, superimposed on a slow depolarization (“calcium”) event. Cells were identified as RS cells if they responded to increasing amplitude of current injection with regular trains of spikes. For a detailed discussion on distinguishing IB and RS cells, see Jacob et al. (2012). In rats. the two L5 cell subtypes are not strictly confined to sublayers 5a and 5b (Nayaranan et al. 2015), but we still observed that two-thirds of IB cells lay within layer 5b. Recordings from fast-spiking neurons were made but not included in this data set.

Our histological methods were designed to recover cell morphology. Therefore, we used a functional assay to identify the principal barrel. At the beginning of every electrode penetration, local field potentials were recorded systematically in L4. The principal whisker was identified as the whisker evoking the shortest latency, fastest rise time-evoked potential in L4. Intracellular recordings were performed within the same electrode penetration only if an obvious principal whisker emerged in the D-row. Septal recording locations are unlikely with this method because evoked potentials in septal locations tend to generate similar responses to several whiskers. In a subset of experiments, the angle of the electrode was slightly off normal to a tangent to the brain surface, and this was taken into account in aiming at the D-row barrels.

#### Chernoff information.

Chernoff information is an objective way to characterize discriminability between two probability distributions. We measured the “distance” between an evoked firing rate trace *f*(*t*) and a baseline firing rate trace *g*(*t*) by using the Chernoff information (Chernoff 1952; Cover and Thomas 1991; Kang et al. 2004), assuming that spiking probability in each condition was approximated by an inhomogeneous Poisson processes. This quantity is an upper bound on the accuracy of an optimal decoder in discriminating whether the observed spikes are generated from the firing rate trace *f*(*t*) or *g*(*t*). We visually inspected the validity of the inhomogeneous Poisson assumption after applying the time-rescaling method (Brown et al. 2002) but found no clear deviation in most cells, possibly because of the low spike count under our sparse stimulation protocol. Notably, the Chernoff information is meaningful even if the inhomogeneous Poisson assumption is violated; in this case, this quantity summarizes the accuracy of a simple decoder based on linear summation of spikes instead of an optimal decoder. *f*(*t*) was the peristimulus time histogram (PSTH) after stimulation of a whisker (bin = 1 ms). *g*(*t*) =*g*_0_ was a constant function whose value was the mean firing rate estimated from 40 to 10 ms before the stimulus onset. The Chernoff information upper bounds the error in estimating the presence or absence of the whisker stimulus on the basis of an observed spike train. In the current context, the Chernoff information is simply approximated by

$$I≅\underset{\alpha }{\max}\left\{-\underset{t}{\sum }\log[{\left(f\left(t\right)\Delta \right)}^{\alpha }{\left(g\left(t\right)\Delta \right)}^{1-\alpha }+{{\left(1-f\left(t\right)\Delta \right)}^{\alpha }}^{\;}{\left(1-g\left(t\right)\Delta \right)}^{1-\alpha }]\right\}\;$$

$$≅\underset{\alpha }{\max}\underset{t}{\sum }\Delta \left[\alpha f\left(t\right)+\left(1-\alpha \right)g\left(t\right)-f^{\;\alpha }\left(t\right)g^{1-\alpha }\left(t\right)\right]$$

when the size of the time bin, Δ, is small enough so that each bin does not contain more than one spike. The maximum of the integral with respect to the parameter α was computed by discretely sampling the range with step size 0.01. If the difference between firing rate traces *f*(*t*) and *g*(*t*) is small, the Chernoff information becomes equivalent to the Fisher information, a measure widely used in neuroscience to quantify detectability of a small parameter change (Seung and Sompolinsky 1993; Toyoizumi et al. 2006). The Chernoff information is more general than the Fisher information because it can also characterize large “distance.” An in-depth explanation about Chernoff information in the neuroscience context is given elsewhere (e.g., Kang et al. 2004).

#### Receiver operating characteristic.

For each stimulus condition, the overlap between the distributions of spike counts in trials with whisker and without whisker stimulation was calculated using receiver operating characteristic (ROC) analysis. The ROC curve was created by plotting the proportion of stimulus trials whose spike counts were above the threshold against the proportion of null trials whose spike counts were above the same threshold. This was repeated for various thresholds. The area under ROC (AUROC) was calculated by measuring the area under the ROC curve. When the area was below 0.5, the area above the curve was taken instead. A larger AUROC indicates that the two distributions are more separated. This was repeated for variable upper limits of the time window in the range of 6 to 60 ms after the stimulus in 1-ms steps. The lower limit of the time window was 5 ms after the stimulus.

#### Initial slope analysis.

The initial slope of the PSPs was defined as the maximum PSP derivative. When the maximum did not correspond to the initial part of the response, the whiskers were excluded from the analysis by visual inspection (29 cells).

#### Latency analysis.

Latency was defined as the first time point following stimulation when the time derivative of the evoked PSP crossed a threshold fixed at mean ± 3 SD beyond the time derivative of the spontaneous activity. After visual inspection, latency was corrected if an obvious false positive was detected (3.8% of the cases). In rare cases, the response was small enough that the derivative of the PSP did not cross the threshold but the response was unambiguously visible. In these cases, the latency was taken as the time of the maximum PSP derivative (1.4% of the cases).

#### Conductance estimation.

Because voltage clamp cannot be achieved with high-access resistance electrodes, we used current-clamp recording for conductance estimation. A range of current injections was used to maintain the cell hyperpolarized or slightly depolarized. We avoided depolarizations that would recruit the nonlinearities of the current-voltage (*I-V*) curve, as recommended by Monier et al. (2008). Receptive fields were mapped during random alternation between three to five different levels of current injection (−40 to +20 pA) that always included the zero-current level. Recorded membrane potential traces were median-filtered with a 10-ms window to eliminate residual spikes. For each whisker, at each time point *t*, a linear regression model [*v*(*t*) = *v*_0_(*t*) + *a*(*t*)*I*, where *v*_0_(*t*) and *a*(*t*) are regression coefficients relating injection current *I* to membrane potential *v*(*t*)] was fit to minimize the sum of squared errors in all recordings of the cell with the whisker stimulus. The regression coefficient trace was smoothed [*ã*(*t*) and *ṽ*(*t*)] and differentiated [*ȧ*(*t*) and *v̇*(*t*)] with respect to time both using the Savitzky-Golay filter. A third-order polynomial was used, and the window length was 5 ms (1 ms for 2 cells with sharp response). The overall results did not change if a 5-ms window was used in all the cells.

First, total conductance and reversal potential were estimated on the basis of the following equations, where *C* is the measured capacitance of the cell:

$$G_{tatal}\left(t\right)=\frac{1}{\overset{˜}{a}\left(t\right)}-C\frac{\overset{․}{a}\left(t\right)}{\overset{˜}{a}\left(t\right)}$$

$$E\left(t\right)={\overset{˜}{v}}_{0}\left(t\right)+C\frac{{\overset{․}{v}}_{0}\left(t\right)}{G_{total}\left(t\right)}$$

Leak conductance (*G*_rest_) and reversal potential (*E*_rest_) were estimated by averaging −40 to −10 ms before the stimulus onset.

Evoked excitatory [*G*_ex_(*t*)] and inhibitory conductances [*G*_in_(*t*)] were estimated by solving the following equation:

$$G_{total}\left(t\right)=G_{ex}\left(t\right)+G_{in}\left(t\right)+G_{rest}$$

$$E\left(t\right)G_{total}\left(t\right)=E_{ex}G_{ex}\left(t\right)+E_{in}G_{in}\left(t\right)+E_{rest}G_{rest}$$

where *E*_ex_ = 0 mV and *E*_in_ = −75 mV are excitatory and inhibitory reversal potential, respectively. Evoked reversal potential (Δ*E*) was estimated by subtracting the baseline average *E*_rest_ from *E*.

#### Statistical tests between control and deprived conditions.

We typically recorded one to two cells of a given type per animal. Whiskers were first categorized in three trimmed whisker classes and six spared whisker classes by sorting their response level. We then tested the effect of deprivation on deprived and spared whiskers with a two-way ANOVA using the aligned rank transform (ART; Wobbrock et al. 2011) with cells as independent samples (the number of cells is indicated by *n*_C_). With ART, ANOVA can be applied without making assumptions about the distribution of the data.

In addition, a bootstrap test was applied to all combinations of a cell and whisker as independent samples (number of whisker-cell pair is indicated by *n*_WC_) for times around the stimulus. The bootstrap test has the advantage of making no assumption about the distribution of data and of being adapted to low sample size. In most cases, the bootstrap method yielded the same level of significance as ART-ANOVA. For each group, the quantity of interest was computed from each of the 1,000 data sets resampled from the corresponding data group, allowing replacement (bootstrap). This provided resampled differences of the quantity of interest, from which we reconstructed a distribution of differences between the groups. The *P* value was then calculated by assuming the null hypothesis that there is no statistical difference between the control and deprived conditions. Hence, a one-tailed *P* value in this scenario is given by the probability that this difference is either positive or negative. The two-tailed *P* value is twice the smallest one-tailed *P* value. The difference in control vs. deprived groups was considered statistically significant if *P* < 0.05. The limits of the confidence interval are defined as 2.5% and 97.5% percentiles of the resampled quantities of interest computed from the group. We tested the difference in mean firing rates (shown in Fig. 2) and the difference in Chernoff information computed on the basis of population average of the PSTHs (shown in Fig. 3). Furthermore, we tested the difference in median membrane potential (shown in Fig. 5) and the difference in median conductance/evoked reversal potential (shown in Fig. 7) to avoid sensitivity of the results to a small number of outliers.

A color bar at the *top* of each panel in Figs. 2–5 and 7 indicates the time at which the population represented by that color is significantly greater than the other population using the bootstrap test (*P* < 0.05). Initial slope and latency distributions shown in Fig. 6 were compared using the Wilcoxon rank sum test or ANOVA.

#### Data representation.

In this article, we characterize population statistics using bootstrap resampling (unless we mention otherwise). For the quantity of interest, we quote the bootstrap median (BM), upper confidence interval (UCI), and lower confidence interval (LCI), which are respectively 50%, 97.5%, and 2.5% percentiles of the bootstrap samples. Likewise, we plot these three percentiles in each panel of Figs. 2–5 and 7.

## RESULTS

**Fig. 1.**
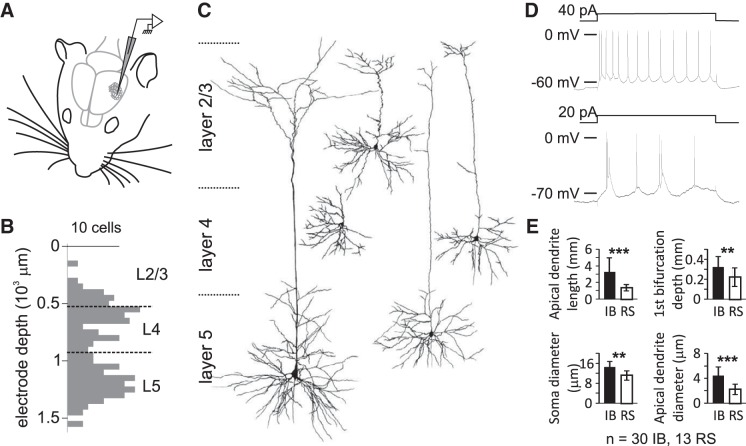
Cortical cell types studied in vivo in the barrel cortex using intracellular recording. *A*: schematic representation of the recording setup. *B*: distribution of the depth below the pia of recorded cells. *C*: dendritic reconstruction of 5 cells filled with biocytin. In L2/3, we only encountered cells with a pyramidal morphology. In L4, we recorded from both spiny stellate (*left*) and pyramidal cells (*right*). Both cell types were present in the sample but not treated separately in the analysis for L4. In L5, we distinguished between thick tufted (*left*) and thin slender (*right*) pyramidal cells. *D*: characteristic responses of cells to somatic current injection: an example of L5RS cell spiking (*top*) and an example of L5IB cell spiking showing the characteristic burst of spikes of decreasing amplitude riding on an envelope of depolarization (*bottom*). *E*: comparison of the morphology of L5 neurons categorized as IB or RS by their spiking characteristics. Note that, on average, IB cells had larger somata and longer, wider dendrites, and their dendrites bifurcated at a greater depth than for RS cells. **P < 0.01; ***P < 10^−4^, Wilcoxon rank sum test.

Intracellular recordings were performed in vivo in the D-row columns of the barrel cortex of juvenile rats (∼P30; Fig. 1*A*). Cortical layers were assessed by the depth from pia (deeper L2/3, mentioned as L3 for simplification, L4, and L5, Fig. 1*B*). A subset of these cells was studied morphologically (Fig. 1*C*). L4 cells (*n* = 5) were categorized into stellate-like or pyramidal-like morphology; L3 cells (*n* = 4) typically had dendritic branching within L2/3; and L5 cells (*n* = 43) were either thick tufted or thin slender pyramidal neurons, as found in earlier studies (Simons and Woolsey 1984; Zhu and Connors 1999). L5 cells were further classified as intrinsically bursting (L5IB) or regular spiking (L5RS) according to their firing pattern in response to current injection, and the classes correlated with their morphology (Fig. 1, *D* and *E*; Jacob et al. 2012).

**Fig. 2.**
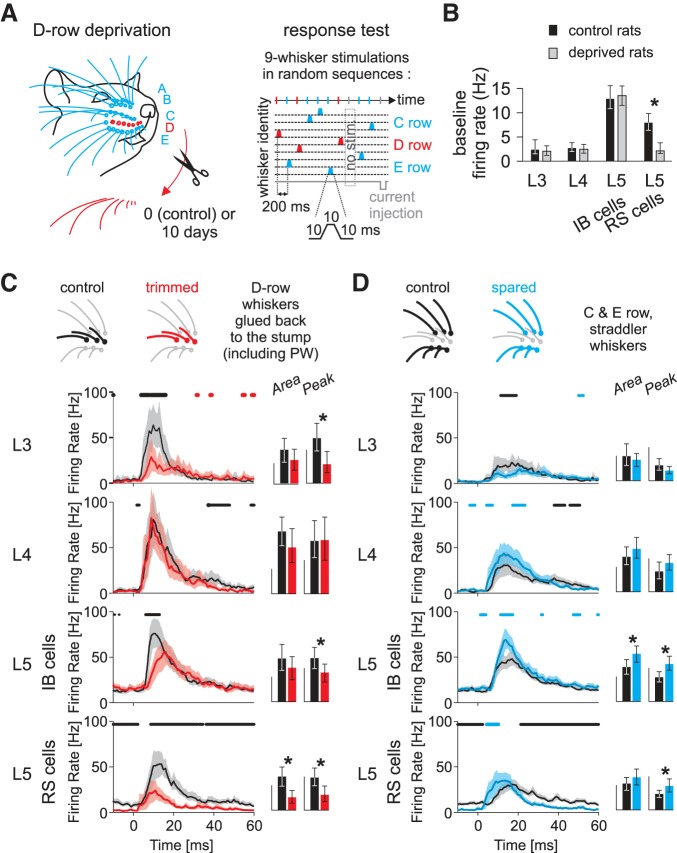
Methodology for measuring the effect of whisker row deprivation on sensory responses in a deprived barrel cortex column. *A*: deprivation protocol. *Left*, the D-row whiskers are trimmed for 10 days, leaving only the whisker stump. The D-row whiskers are glued to the stump before mechanical stimulation; for the control case, D-row whiskers are trimmed and then glued immediately. *Right*, schematic representation of a stimulation sequence. Receptive fields are tested with a 9-whisker stimulator arranged in a 3 × 3 grid with the deprived D-row (red) at the center. Each line represents the position of a whisker as a function of time. One stimulation sequence comprises *1*) a random permutation of stimulations of each of the 9 whiskers (colored trapezoids) and a time event without stimulation (gray rectangle) and *2*) an intracellular injection of current at the end to test cell resistance (*bottom* line). Inter-whisker intervals and an expanded trace to show the time course of the stimulus (*bottom inset*) are indicated. *B*: plasticity of baseline firing rate. Bars and error bars show the median and 95% confidence interval (bootstrap). Note that only the L5RS cells change their baseline firing rate in response to deprivation. *C*: plasticity of response to trimmed whiskers. Drawings at *top* are schema showing the positions of the whiskers included in the analysis (control, black; deprived, red). Population PSTHs of the response to trimmed whisker stimulation are plotted for each category of cell. The line shows the average firing rate, and the shaded area represents the 95% confidence interval (bootstrap). For each time point, control and trimmed whisker responses are compared using statistical test (bootstrap test). The horizontal bars above the graphs indicate the time points for which there is a significant difference (*P* < 0.05, bootstrap test) between the time plots (depression, black; potentiation, red). At *right*, the bar charts show the area (calculated after subtracting spontaneous activity; scale bar, 0.5 spike/stimulus) and peak (scale bar, 50 Hz) of the population PSTHs; error bars show 95% confidence intervals (bootstrap). *D*: plasticity of response to the spared whiskers recorded in the deprived barrel column. Conventions are the same as in *C* except that potentiation and spared whiskers are coded blue. *P < 0.05, bootstrap test.

Sensory deprivation was induced by trimming the D-row whiskers (Fig. 2*A*). After 10 days of deprivation, trimmed whiskers were re-glued on the whisker stump for testing cortical responsiveness. Responsiveness was compared with that in control animals, whose whiskers were trimmed and re-glued on the day of recording. To assess changes in the receptive fields of recorded cells rapidly and automatically, we used a whisker stimulator containing 9 piezoelectric bimorph wafers attached to 9 whiskers in a 3 × 3 square grid such that the principal whisker was located at the center of the grid (Fig. 2*A*). We applied a pseudorandom sequence to stimulate the whiskers, comprising 50 stimuli for each of the 9 whiskers plus a null period of no stimulation, which we used to estimate spontaneous activity (Jacob et al. 2008, 2012). Evoked activity was evaluated by subtracting spontaneous activity and averaging the response over 50 stimuli. Overall, the absolute level of spontaneous and evoked activity reported (Fig. 2) cannot be directly compared with that of other studies due to the use of sharp microelectrodes. However, the relative incidence of each cell type is in agreement with the literature (de Kock et al. 2007).

### 

#### Effect of deprivation on spontaneous activity.

Spontaneous activity was unchanged by deprivation in all but the L5RS cells (Fig. 2*B*). After whisker deprivation, the mean spontaneous firing rate of L5RS cells located in the deprived row decreased approximately fourfold in row-deprived animals (control: BM = 7.9 Hz, deprived: BM = 2.1 Hz). This difference was highly statistically significant (*P* < 0.001, bootstrap test) and can be seen in the prestimulus period of the PSTHs (Fig. 2, *C* and *D*, L5RS). Spontaneous activity was unchanged for any other cell type studied, including L5IB cells and cells located in other layers (see Fig. 2 and Table 1).

**Table 1.** Spiking properties

	Control				Deprived				
Layer (Spared/Trimmed)	No. of cells	Mean	Lower 95% CI	Upper 95% CI	No. of cells	Mean	Lower 95% CI	Upper 95% CI	*P* Value
*Total evoked (3–103 ms), spikes/stimulus*									
L3 (T)	10	0.677	0.439	0.896	13	0.482	0.254	0.69	0.00760*
L3 (S)	10	0.495	0.302	0.746	13	0.417	0.289	0.557	0.4795
L4 (T)	14	1.24	0.968	1.55	12	0.932	0.601	1.32	0.19686
L4 (S)	14	0.71	0.541	0.889	12	0.866	0.681	1.1	0.1124
L5IB (T)	27	0.864	0.62	1.13	22	0.684	0.45	0.913	0.06672
L5IB (S)	27	0.692	0.538	0.848	22	0.959	0.792	1.13	0.00946*
L5RS (T)	23	0.685	0.485	0.887	21	0.25	0.127	0.4	0.00022*
L5RS (S)	23	0.529	0.389	0.658	21	0.654	0.513	0.823	0.21064
*Baseline (−40 to −10 ms), Hz*									
L3	10	2.35	1.54	4.37	13	2.09	1.26	3.24	0.65
L4	14	2.69	1.98	3.74	12	2.47	1.67	3.45	0.701
L5IB	27	12.9	10.8	15.4	22	13.5	11.6	15.6	0.683
L5RS	23	7.93	6.25	9.8	21	2.14	1.6	3.8	0*

Suprathreshold responses of neurons in different layers of the cortex to stimulation of the whiskers are tabulated along with the spontaneous firing rates. The mean firing rate for evoked responses are estimated over the period 3–103 ms poststimulus after subtracting spontaneous activity. The spontaneous firing rate period is estimated over −40 to −10 ms before the stimulus (Hz). Upper and lower 95% confidence intervals are calculated using the bootstrap method. *P* values compare control and deprived values using the aligned rank transform (ART) ANOVA test (evoked) or bootstrap test (baseline).

* *P* < 0.05. T refers to trimmed whisker and S to spared whisker stimulation.

#### Effect of deprivation on evoked activity.

Evoked activity was affected by deprivation, and the amplitude and direction of the effect depended on cell type, layer location, and the identity of the stimulated whisker (trimmed or spared, Fig. 2, *C* and *D*). For trimmed whiskers, the mean firing rate decreased to 71% of control levels in L3 (*P* = 0.008, ART-ANOVA). In L5, the mean firing rate of RS cells decreased to 36% of control levels (*P* = 0.0002) The mean firing rate evoked by trimmed whiskers was unaffected by deprivation for cells located in L4 (*P* = 0.20) and for IB cells in L5 (*P* = 0.07). In summary, trimming the whiskers led to lower trimmed whisker responses in L3 and L5RS cells but not in L4 and L5IB cells, and the magnitude of the decrease was largest for L5RS cells (Table 1).

For spared whisker responses, the mean firing rate of L5IB cells increased to 139% of control values, which was statistically significant (*P* = 0.009). However, no other cell types showed changes in spared whisker responses (L3, *P* = 0.48; L4, *P* = 0.11; L5RS, *P* = 0.21) as shown in Fig. 2 and Table 1.

#### Effect of deprivation on sensory information coding.

**Fig. 3.**
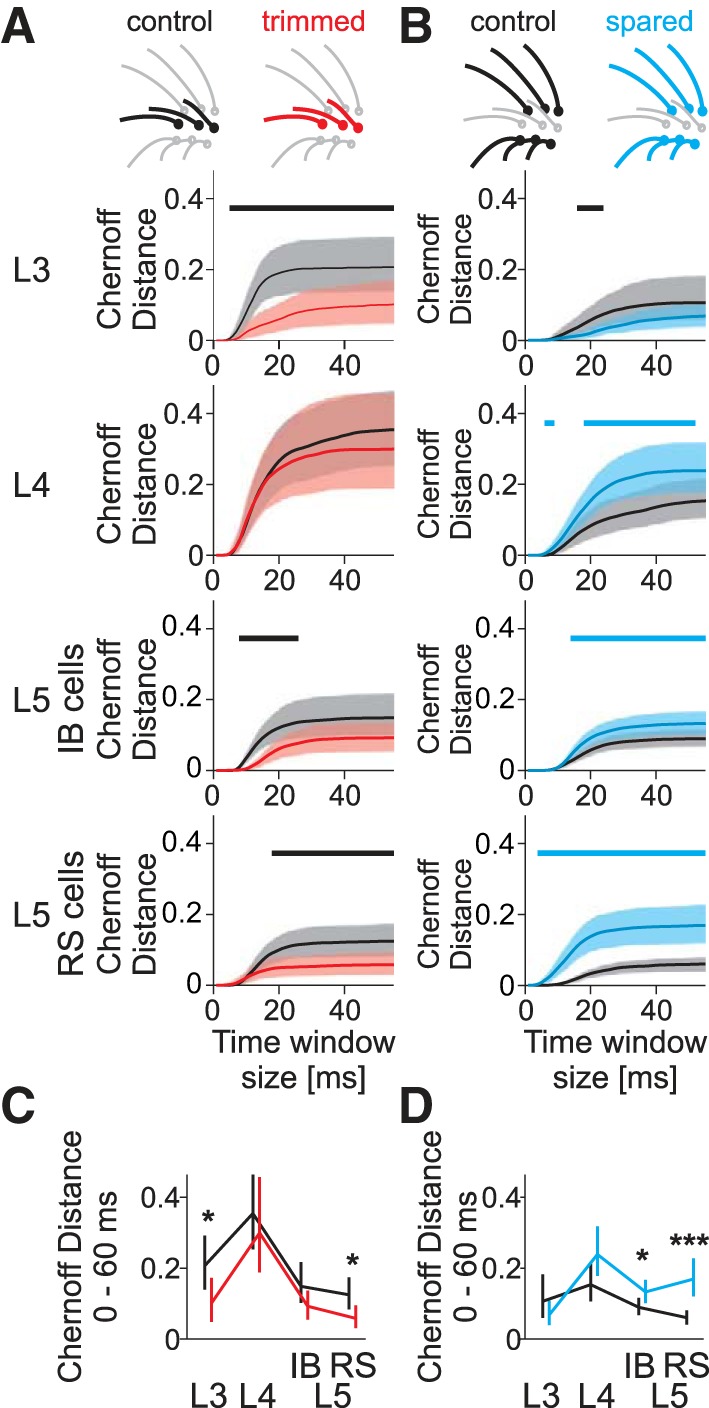
Chernoff information contained in spike trains as a measure of plasticity of sensory responses. *A*: trimmed whiskers. For the different layers and cell types, Chernoff information between spontaneous activity and population PSTH is plotted as a function of increasing time window sizes starting at *time 0* after stimulation. The horizontal bar above each plot indicates the time at which a significant increase (blue) or decrease (black) in Chernoff information occurs due to sensory deprivation (bootstrap test). *B*: spare whiskers. Chernoff information is plotted for cells in each layer using the same conventions as in *A*. Note that potentiation occurs in all layers except L2/3. *C*: summary of median Chernoff information for the largest window (0–60 ms) as a function of cell types for trimmed whiskers. Error bars represent 95% confidence interval. *D*: same conventions as in *C* but for spared whiskers. *P < 0.05, ***P < 0.001, bootstrap test.

The information a neuron receives about a stimulus depends in part on differences between the evoked and spontaneous activity. The spontaneous activity of L5RS cells was reduced after deprivation, which suggested that information might change even if spared whisker responses remained constant. Therefore, we investigated deprivation-induced changes in information coding across cortical layers and cell types. We quantified the Chernoff information (Chernoff 1952; Cover and Thomas 1991; Kang et al. 2004), which summarizes detectability of whisker stimulation on the basis of population responses (shown in Fig. 3). For each cell type, the Chernoff information measures a distance between the time-varying population firing rate under evoked and spontaneous conditions, assuming inhomogeneous Poisson spiking of neurons (see methods). Although sensory information is generally coded by more than just firing rate (de Ruyter van Steveninck et al. 1997), including pattern of firing such as bursts, such extra features were not prominent in our data, possibly because of the relatively low spiking activity and the sparse stimulation protocol applied in this study. Chernoff information was calculated as accumulating with each 1-ms time bin from 1 to 60 ms poststimulus. Aggregate Chernoff information about the stimulus was contained in the last time window (60 ms) and for each response appeared to reach an asymptote by 60 ms (see Fig. 3). To obtain the Chernoff information in an unbiased way based on rare spiking events, we calculated Chernoff information of the population mean responses rather than the single-cell responses. However, the results were qualitatively similar when the information was calculated for individual cells (data not shown). This result suggests that the variability of sensory response in the current experiment was relatively small within each cell type and emphasizes the robust nature of the finding.

In control undeprived animals, Chernoff information was maximal in L4 cells. Whiskers in the same row as the principal whisker, corresponding to the trimmed row in deprived animals, had bootstrapped Chernoff information of BM = 0.36 [UCI = 0.47, LCI = 0.26, *n*_WC_ = 40 (14 cells)]. In the flanking rows, which are spared in the deprived animals, Chernoff information reached median BM = 0.15 [UCI = 0.22, LCI = 0.11, *n*_WC_= 77 (14 cells)]. In control animals, Chernoff information for both flanking and same-row whiskers decreased in the order L4 > L3 > L5IB > L5RS.

For all cell types except L5RS cells, the effect of whisker deprivation on Chernoff information matched the changes in evoked firing rate. After deprivation, Chernoff information was unchanged in L4, which retained the greatest information in the column (as with undeprived control animals). For the deprived-row whiskers, Chernoff information was significantly decreased in L3 and L5RS cells [L3: control BM = 0.21, *n*_WC_ = 30 whisker cell pairs (10 cells), deprived BM = 0.11, *n*_WC_ = 36 (13 cells), *P* = 0.030, bootstrap test; L5RS: control BM = 0.13, *n*_WC_ = 60 (23 cells), deprived BM = 0.058, *n*_WC_ = 58 (21 cells), *P* = 0.018, bootstrap test] and delayed in L5IB cells, but the order by which information decreased by layer remained unchanged.

The situation was different for the spared whisker responses. In L4 cells, Chernoff information was not significantly increased, though it was advanced in time (Fig. 3). In L5 cells, Chernoff information was significantly increased (L5IB spared: *P* = 0.032, L5RS spared: *P* < 0.001, bootstrap test) and in addition, the increase in information was substantially advanced in time for the L5RS cells. Finally, there was no significant change in spared whisker information in L3 (*P* = 0.27). As a consequence, the ordering of information for spared whisker stimulation was reorganized by sensory deprivation such that activity in L5RS cells contained almost as much Chernoff information as in L4 cells (BM = 0.17 vs. 0.24, respectively) and far more than in L3 and L5IB cells (BM = 0.070 and 0.133, respectively). Therefore, sensory deprivation changed the order of information representation for spared whiskers within a deprived barrel column to L4 > L5RS > L5IB > L3.

**Fig. 4.**
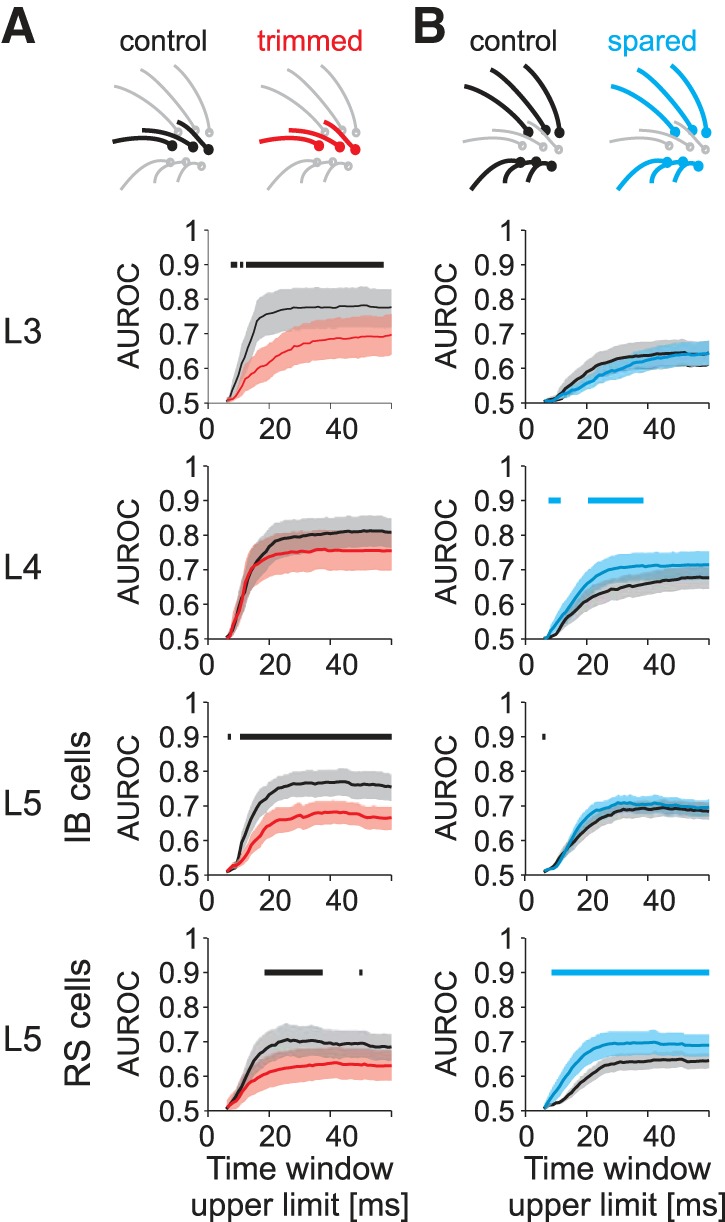
Area under receiver operating characteristic curve (AUROC) calculated independently for each cell and whisker. *A*: trimmed whiskers. For the different layers and cell types, AUROC between distributions of spike counts in trials with whisker stimulation and in null trials without stimulation is plotted as a function of increasing time window upper limit starting at time 6 ms after stimulation. Time window lower limit is 5 ms after stimulation. The horizontal bar above each plot indicates the time at which a significant increase (blue) or decrease (black) in mean AUROC occurs due to sensory deprivation (bootstrap test). *B*: spare whiskers. AUROC is plotted for cells in each layer using the same conventions as in *A*. Shaded areas represent the 95% confidence interval.

Although Chernoff information takes into account changes in the time course of neural response, we also used a simpler ROC analysis based on spike count to check the robustness of the finding (Fig. 4). The results of the ROC analysis and the Chernoff information analysis were very similar in that the effect of whisker trimming produced the same direction of change in each case. The only difference was that the spared whisker response of L5IB cells did not show a significant difference under ROC analysis despite the increased spike count. In contrast, L5RS cells, which were of particular interest, showed a robust increase in sensory detection performance by either measure.

#### Plasticity of subthreshold responses.

**Fig. 5.**
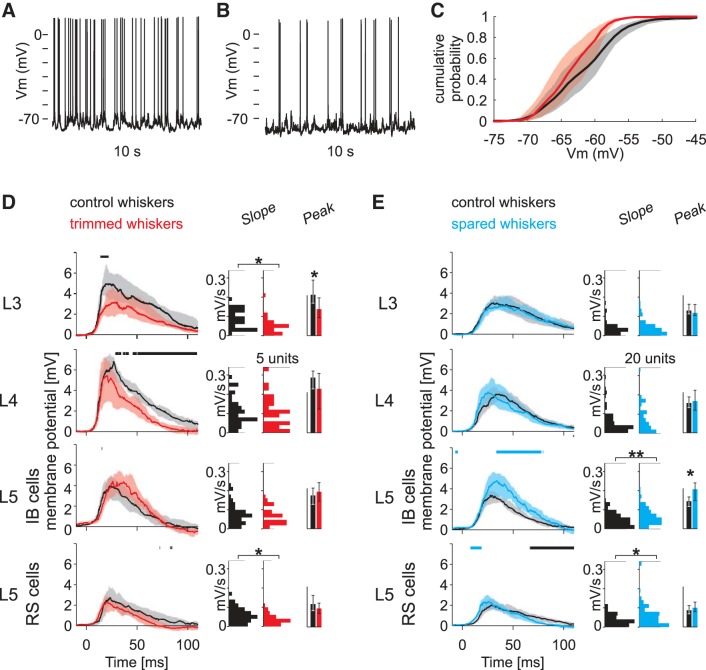
Plasticity of membrane potentials (*V*_m_) and postsynaptic potentials (PSPs) along the barrel cortex column. *A*: 10 s of ongoing activity for a L5RS cell in a control rat. *B*: 10 s of ongoing activity for a L5RS cell in a deprived rat. *C*: for each L5RS cell, cumulative density of *V*_m_ during the blank stimuli was calculated. For statistical robustness, outlier cells were removed (2 cells with the lowest median *V*_m_ value and 2 cells with the highest median *V*_m_ value for both deprived and control). Thick line shows the mean *V*_m_ cumulative distribution for control (black) and deprived (red) cells. Shaded areas delineate the bootstrap confidence interval (95%). *D*: trimmed whiskers. For the different layers and cell types, median tPSPs are plotted. The horizontal lines above the graphs indicate the time points for which there is a significant difference (*P* < 0.05, bootstrap test) between control and trimmed whiskers (depression, black; potentiation, red). At *right*, distribution of tPSP initial slope is plotted as a histogram (scale bar, 5 units). The bar charts represent area and peak of median tPSPs and 95% confidence interval (scale bars, 0.2 s·mV for area and 5 mV for peak). *E*: spared whiskers. Averaged sPSPs are plotted for the different layers and cell types. Conventions are the same as in *D* except that potentiation is coded blue and the scale bar for initial slope distribution is 20 units. *P < 0.05, **P < 0.001, bootstrap test.

To explore neuronal mechanisms underlying the deprivation-induced changes in sensory information representation, we analyzed membrane potential in each group of cells. First, we tested for differences in “resting” membrane potential of cells in different layers (Table 2). We found that there was no difference in mean resting membrane potential for cells in L3, L4, and L5IB but that L5RS cells showed a significant hyperpolarization from −60.7 to −65.6 mV in deprived animals (*P* < 0.006, bootstrap test). This is consistent with the significant decrease in spontaneous action potential firing rate for L5RS cells (Table 1). Figure 5, *A–C*, shows example traces and distribution of ongoing membrane potential activity for L5RS cells with and without whisker deprivation. Deprivation did not induce a global shift of the membrane potential distribution toward hyperpolarization, but rather induced greater losses for depolarized membrane potential values.

**Table 2.** Membrane potential

	Control				Deprived				
Layer (Spared/Trimmed)	No. of cells	Median	Lower 95% CI	Lower 95% CI	No. of cells	Median	Lower 95% CI	Upper 95% CI	*P* Value
*Peak membrane potential, mV*									
L3 (T)	14	4.94	4.16	6.87	13	3.23	2.33	4.8	0.03578*
L3 (S)	14	3.11	2.71	3.84	13	2.81	2.47	3.82	0.87248
L4 (T)	15	6.81	5.43	7.63	12	5.44	2.92	7.35	0.24845
L4 (S)	15	3.68	3.11	4.59	12	3.9	2.67	5.27	0.86453
L5IB (T)	27	4.04	3.09	4.99	22	4.57	3.38	5.8	0.71907
L5IB (S)	27	3.39	2.71	3.99	22	4.89	3.48	5.75	0.00048*
L5RS (T)	23	2.83	2.03	3.91	21	2.3	1.69	2.99	0.26878
L5RS (S)	23	2.08	1.63	2.68	21	2.43	1.85	3.08	0.41965
Layer	No. of cells	Mean	Lower 95% CI	Upper 95% CI	No. of cells	Mean	Lower 95% CI	Upper 95% CI	*P* Value
*Resting membrane potential, mV*									
L3	14	−61.35	−58.35	−64.57	13	−64.90	−61.33	−68.99	0.162
L4	15	−66.30	−62.03	−71.74	12	−63.02	−59.33	−66.56	0.122
L5IB	27	−63.69	−60.98	−66.30	22	−65.39	−62.63	−68.52	0.052
L5RS	23	−60.74	−58.45	−63.20	21	−65.55	−62.57	−68.54	0.006*

Membrane potential responses to stimulation of the whiskers (with spikes removed) are tabulated along with the resting membrane potential for neurons in different layers of the cortex. Median membrane potentials for evoked responses and means for resting potential are shown together with the upper and lower 95% confidence intervals, which are calculated using the bootstrap method. *P* values compare control and deprived values using the ART-ANOVA test (peak) or bootstrap test (resting).

* *P* < 0.05. T refers to trimmed whisker and S to spared whisker stimulation.

Next, we analyzed the trimmed whisker-evoked postsynaptic potentials (tPSPs) and the spared whisker-evoked postsynaptic potentials (sPSPs) in L3, L4, L5IB, and L5RS cells (Fig. 5, *D* and *E*, Table 2). For L3 cells, whisker deprivation produced depression of trimmed whisker responses measured by peak of median tPSP, which decreased to 65% of control values (*P* = 0.04, ART-ANOVA), and initial slope of tPSPs, which decreased to 54% of control values [control: median = 0.84 V/s, *n*_WC_ = 35 (12 cells); deprived: median = 0.46 V/s, *n*_WC_ = 36 (13 cells); *P* = 0.015, Wilcoxon's rank sum test]. For L4 cells, we observed no changes in the peak tPSP (*P* = 0.25, ART-ANOVA) or tPSP slope (*P* = 0.63, Wilcoxon's rank sum test).

For L5 cells, tPSP responses were not significantly affected in general, except for a significant slope reduction for L5RS cells (*P* = 0.02, Wilcoxon's rank sum test). Together with the hyperpolarization of resting membrane potential in this population following the deprivation, this naturally accounts for the deprivation-induced decrease in the firing rate response to trimmed whiskers in L5RS cells. Conversely, for L3 cells, the decrease in firing rate caused by deprivation is directly correlated with a decrease in membrane potential depolarization.

For spared whiskers responses, L3 and L4 sPSPs were unchanged overall after deprivation. However, sPSPs were increased in L5IB cells as judged by the peak of the median sPSP, which increased to 144% of control values (*P* = 0.0005, ART-ANOVA), and the sPSP initial slope, which increased to 132% of control [control: median = 0.44 V/s, *n*_WC_=111 (19 cells); deprived: median = 0.58 V/s, *n*_WC_ = 98 (16 cells), *P* < 0.001, Wilcoxon's rank sum test]. Finally, for L5 RS cells, only the slope of the sPSPs was increased to 139% [control: median = 0.32 V/s, *n*_WC_ = 80 (17 cells); deprived: median = 0.44 V/s, *n*_WC_ = 106 (17 cells), *P* = 0.014, Wilcoxon's rank sum test], but not the peak of the sPSP (Table 2). This sharper rise of sPSPs after whisker deprivation is consistent with the shorter latency spiking response to spared whiskers in this population. We explore later whether this change involves changes in excitatory and inhibitory synaptic conductances.

#### Plasticity of response latency.

**Fig. 6.**
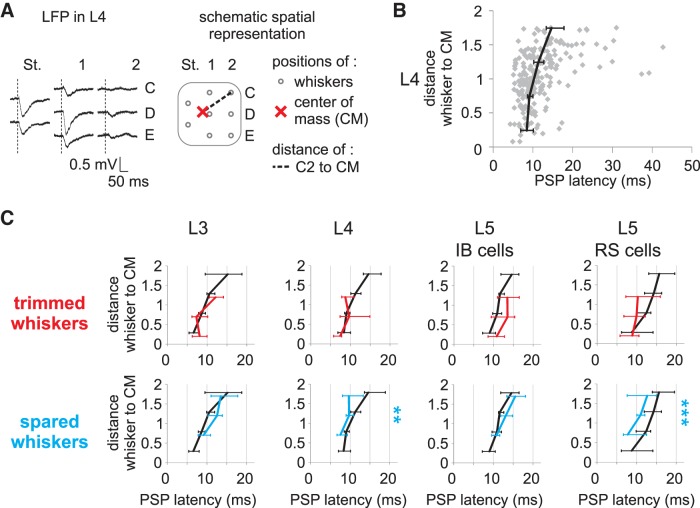
Plasticity of PSP latencies along the barrel cortex column. *A*: methods for calculating the spatial distance between each whisker and center of mass (W minus CM) and PSP latencies. Local field potentials (LFPs) were recorded in L4 with the same electrode and at the same penetration used for intracellular recordings. *Left*, example of LFP responses to 8 different whiskers. The initial slope of the negative potential is used to calculate the CM of the receptive field. *Right*, schematic representing the position of the whiskers (gray circles) and the CM (red cross) and the distance between whisker C2 and the CM (dashed line) in a Cartesian whisker space. In the whisker space, distance between rows and between arcs is arbitrarily set to 1. The straddler's positions (delta and gamma) are set at halfway between the two rows. *B*: distribution of PSP latencies for L4 recordings. The distance between whisker and the L4 LFP CM is plotted against PSP latencies (gray diamonds). Distances are grouped in 0.5 intervals of whisker space to calculate the median (black lines). Error bars show the 95% confidence interval (bootstrap). *C*: plasticity of latencies. For each 0.5 distance interval, medians of PSP latencies are plotted for control responses (black), trimmed whisker responses (red), and spared whisker responses (blue). Error bars show the 95% confidence interval (bootstrap). Because recordings were always in the deprived column, the distance W minus CM was never larger than 1.5 for trimmed whiskers and never smaller than 0.5 for spared whiskers. Significant differences between control and deprived cases are tested with Wilcoxon's rank sum test. Note the highly significant latency shifts for L4 and L5RS spared whisker responses (**P < 0.01; ***P < 0.001).

The relative latencies recorded in the different groups of neurons provide additional information on how sensory signals are sent to the cortex and how they travel through layers. The latency of response to remote surround whisker stimulation is much greater than the latency of response to whiskers positioned closer to the center of the receptive field (Armstrong James and Fox 1987), and its estimation is more sensitive to noise. Therefore, we considered latency independently for each whisker rather than averaging all trimmed and all spared whiskers as described above. To study the plasticity of PSP latencies, we plotted latencies against the Euclidian distances between the corresponding whisker positions and a center of mass (CM) of the multiwhisker receptive field, which is evaluated using the coordinates of the whisker positions on the face. To estimate the CM independently from sensory deprivation, we used the CM of evoked local field potentials recorded in L4 during the same electrode penetration (Fig. 6*A*). The selected recording sites had one dominant principal whisker in the D-row that largely defined the center of the receptive field, but it could be offset by secondary inputs from surrounding whiskers. As expected, both latency and variability of latency distribution increased with the distance of the whisker from the receptive field center (Fig. 6*B*, for all cell types, *P* < 0.01, ANOVA).

In the control condition, latency also increased from L4 and L3 to L5 (respectively for L3, L4, L5IB, and L5RS cells: median latency = 9.6, 10.3, 11.4, and 13.5 ms; UCI = 10.3, 11, 12.1, and 14.6 ms; LCI = 9, 9.9, 11.1, and 12.4 ms). In the deprived condition (Fig. 6*C*), the latency of trimmed whisker tPSPs was unchanged for all layers. For spared whiskers, the latency of sPSPs decreased significantly in the entire L5RS cell's receptive field (*P* < 0.001, ANOVA) and in L4 mostly away from the receptive field center (*P* < 0.01, ANOVA). Latencies did not change in L3 and L5IB cells. Interestingly, the latency of sPSPs in L5RS cells (median = 10.5 ms, UCI = 12.4 ms, LCI = 9.2 ms, *n*_WC_ = 83) was shorter than that in L3 neurons (median = 12.3 ms, UCI = 13.4 ms, LCI = 10.6 ms, *n*_WC_ = 80, *P* = 0.05, ANOVA) and L5IB cells (median = 13.2 ms, UCI = 14.4 ms, LCI = 12.1 ms, *n*_WC_ = 88, *P* < 0.01, ANOVA) but was not different from that in L4 neurons (median = 9.3 ms, UCI = 10.7 ms, LCI = 8.7 ms, *n*_WC_ = 65, *P* = 0.13, ANOVA). Moreover, latency of the 10% most rapid sPSPs was significantly shorter in L5 RS cells (median = 5.2 ms) than in L3 cells (median = 7 ms, *P* < 0.001, ANOVA) and in L5IB cells (median = 8.4 ms, *P* < 0.001, ANOVA). This result suggests that short-latency potentiation in L5RS cells is unlikely to be derived either from L3 or L5IB cells.

#### Conductance analysis for L5 cells.

**Fig. 7.**
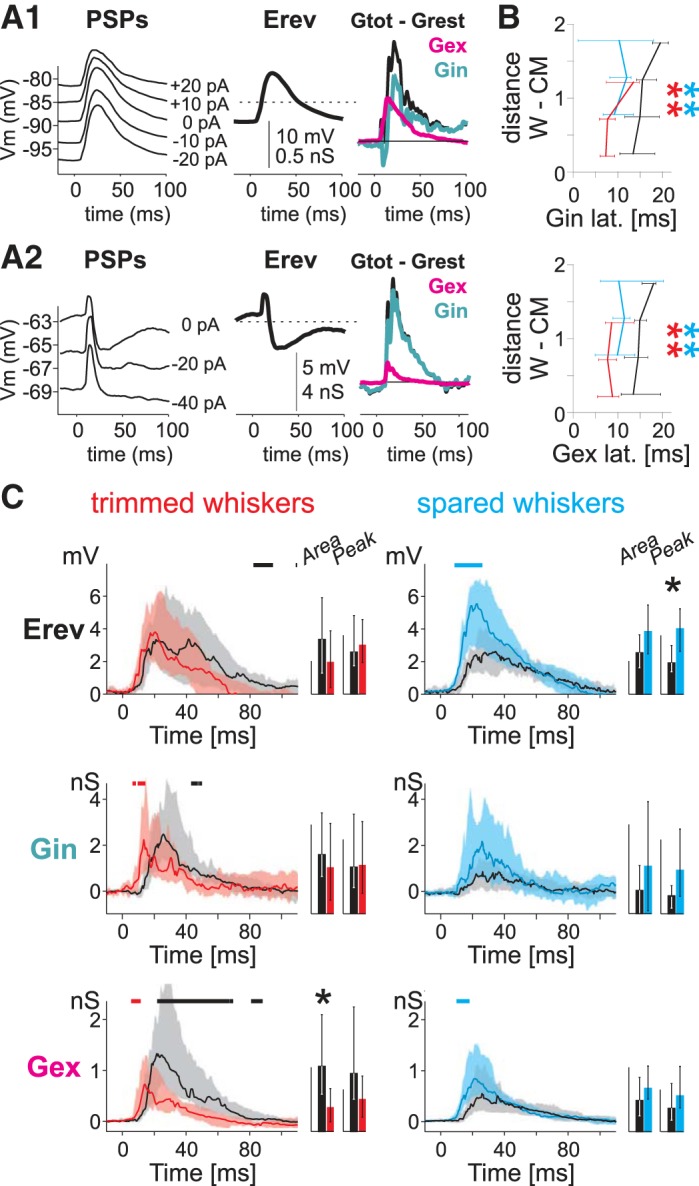
Plasticity of excitatory and inhibitory conductances in L5RS cells. *A1*: evoked conductances and reversal potential calculated on a control L5RS cell. *Left*, PSPs were calculated for different levels of injected currents. *Middle*, reversal potential (*E*_rev_). *Right*, total (*G*_rest_ subtracted) excitatory (*G*_ex_) and inhibitory (*G*_in_) conductances. *A2*: evoked conductances and resting potential calculated on a control L5IB cell. Same conventions as in *A1*. *B*: plasticity of latency of inhibitory (*top*) and excitatory (*bottom*) conductances to control (black), trimmed (red), and spared whiskers (blue). Same conventions as Fig. 6*C*. **P < 0.01, Wilcoxon rank sum test. *C*: plasticity of reversal potential (*top*), inhibitory (*middle*), and excitatory (*bottom*) conductances. Same conventions as Fig. 5, *D* and *E*. The bar charts represent areas (scale bars: *E*_rev_, 0.1 s·mV; *G*_in_, 0.1 s·nS; *G*_ex_, 0.04 s·nS) and peaks (scale bars: *E*_rev_, 5 mV; *G*_in_, 4 pS; *G*_ex_, 1 nS). Error bars indicate 95% confidence interval. *P < 0.05, bootstrap test.

To gain some insight into the excitatory and inhibitory components of whisker responses and their plasticity, we repeated sets of stimuli under various levels of injected current to calculate synaptic conductance and reversal potential (Fig. 7*A*). We recorded conductance for L5 receptive field responses in control and deprived animals. We found that the decrease of latency observed in L5RS cells was associated with both excitatory (*P* < 10^−4^ for trimmed whiskers and *P* < 10^−6^ for spared whiskers, Wilcoxon's rank sum test) and inhibitory components (*P* < 10^−6^ for trimmed whiskers and *P* < 10^−5^ for spared whiskers; Fig. 7*B*).

For trimmed whisker responses, even though the median excitatory and inhibitory conductance peaks were both decreased (Fig. 7*C*; *P* = 0.018 for excitatory conductance and *P* = 0.018 for inhibitory conductance, *n*_C_ = 6 cells, ART-ANOVA), the effects largely canceled one another out to produce no net change in the reversal potential over the same time interval.

For spared whisker responses, both excitatory (*g*_E_) and inhibitory (*g*_I_) conductance peaks increased significantly (*P* = 0.003 and *P* = 0.03, respectively, ART-ANOVA). The early part of the median excitatory conductance waveform from 10 to 17 ms was the main source of the effect (*P* < 0.05, bootstrap test; Fig. 7*C*). In addition, the median reversal potential was significantly increased during a similar period from 9 to 26 ms (*P* < 0.05, bootstrap test), and the peak reversal potential was 209% greater in deprived compared with control animals (Table 3: *P* = 0.026, bootstrap test; *P* = 0.007, ART-ANOVA). Both observations confirmed a stronger increase of excitatory conductance than of inhibitory conductance. Because the earliest part of the subthreshold waveform is related to the period when spikes are produced in response to whisker stimulation, the increase in reversal potential during the first 30 ms of the response most likely explains the increase in spike firing to the spared whiskers during this period (see above on RS cell suprathreshold responses).

**Table 3.** Membrane conductance

	Control				Deprived				
Layer (Spared/Trimmed)	No. of cells	Median	Lower 95% CI	Upper 95% CI	No. of cells	Median	Lower 95% CI	Upper 95% CI	*P* Value
*Excitatory conductance, nS*									
L5IB (T)	6	0.823	0.696	3.44	3	0.98	0.37	1.94	0.2614
L5IB (S)	6	0.701	0.465	0.958	3	1.23	0.487	2.18	0.081611
L5RS (T)	6	1.38	0.78	2.96	6	0.771	0.342	1.31	0.018267*
L5RS (S)	6	0.55	0.268	1.15	6	0.842	0.557	1.55	0.0033439*
*Inhibitory conductance, nS*									
L5IB (T)	6	2.62	1.72	9.1	3	0.859	0.441	5.62	0.59498
L5IB (S)	6	0.94	0.602	1.37	3	1.65	0.911	4.08	0.092052
L5RS (T)	6	2.49	1.39	5.36	6	2.67	1.11	4.97	0.018054*
L5RS (S)	6	0.933	0.314	1.5	6	2.31	0.866	4.55	0.028516*
*Reversal potential, mV*									
L5IB (T)	6	3.88	2.9	5.45	3	4.99	3.28	12.4	0.62465
L5IB (S)	6	4.66	3.11	5.74	3	5.83	4.1	7.86	0.19963
L5RS (T)	6	3.49	2.41	6.66	6	4.19	2.67	6.3	0.7624
L5RS (S)	6	2.66	1.85	4.14	6	5.57	3.6	7.4	0.0071428*
*Input resistance, MΩ*									
L5IB	6	283	126	354	3	150	114	549	0.587
L5RS	6	188	113	324	6	175	98	258	0.730

Excitatory and inhibitory membrane conductances during responses to stimulation of the whiskers are tabulated along with the reversal membrane potential for neurons in L5 of the cortex. Input resistances are also indicated. The median peak of conductances and reversal potentials is shown together with the upper and lower 95% confidence intervals, which are calculated using the bootstrap method. *P* values compare control and deprived values using the ART-ANOVA test.

* *P* < 0.05. T refers to trimmed whisker and S to spared whisker stimulation.

For L5 IB cells, we found no significant difference in the level or latency of total conductance with deprivation. Although all whisker responses showed a trend toward more positive reversal potentials favoring excitatory conductance (data not shown), none were significant (Table 3).

## DISCUSSION

We found that plasticity induced by row deprivation alters the distribution of sensory information within and between the different layers of the cortical columns. To summarize, no major changes were observed in L4 cells except for a decrease in latency to the spared whiskers; the only change we observed in L3 cells was a decrease in response to trimmed whiskers; L5IB cells underwent a small depression to trimmed whisker inputs and a global potentiation to spared whiskers; and finally, L5RS cells exhibited a remarkable form of plasticity resulting in the largest change in information that we observed. L5 cells showed a decrease in spontaneous activity, the potentiation of a short latency excitatory input from the spared whiskers and a decreased response to the trimmed whiskers. We discuss below some of the substrates and pathways for L5 plasticity that was clearly distinct from the more conventional effects observed in L4 and L3.

### 

#### Pathways underlying short-latency responses in L5.

There is a long history documenting the short-latency thalamic ventral posteromedial nucleus (VPm) input to L5 of the somatosensory cortex. Electron microscopy studies have shown that VPm projections make contact with cells in all layers of the barrel cortex except L2, and in particular on L5 (White 1978; White and Hersch 1982). Short-latency whisker responses, concurrent with those in L4, were observed in L5b almost a quarter-century ago (Armstrong-James et al. 1992), and in vitro studies pioneering the thalamocortical slice preparation concurrently demonstrated VPm input to L5 cells (Agmon and Connors 1992). More recent studies have further documented the presence of thalamic input to L5 in both the mouse and rat (Constantinople and Bruno 2013; Petreanu et al. 2009; Rah et al. 2013; Wright and Fox 2010). In the present study we found that an early component of the RS cells response to whisker stimulation was potentiated by row deprivation and that potentiation was related in time to an increase in an excitatory conductance. Given the latency of the response comes before any other cortical cell responds except those in L4 of the principal barrel, the most likely source of the excitation is a direct input from VPm. At present, it is not clear why short-latency input should potentiate in RS cells and not in IB cells. The original report of Agmon and Connors (1992) suggested that IB cells do not receive thalamic input whereas RS cells do. However, there is evidence of the opposite in the auditory cortex (Sun et al. 2013) and in the barrel cortex (Bureau et al. 2006).

For the thalamic input to account for the increased short-latency component generated by spared whisker stimulation, the VPm input would need to convey excitation to a nonprincipal whisker barrel. There are two possibilities: first, branches of thalamic afferents innervating their principal barrels might grow into the neighboring deprived barrel (Oberlaender et al. 2012) during the 10-day deprivation period; second, the nonprincipal whisker inputs that potentiate might already exist, and there is evidence for this view. Blocking intracortical activity with muscimol and locally disinhibiting the recorded cell reveals double-whisker responses or multiwhisker responses in some L5 cells (Wright and Fox 2010).

Potentially, the short-latency input to L5 RS cells might arise from L4 in the neighboring barrel. There is functional evidence from paired recordings, glutamate uncaging, and optogenetic stimulation studies that L4 projects to L5 (Feldmeyer et al. 2005; Petreanu et al. 2009; Schubert et al. 2001). Furthermore, anatomical studies show axons projecting ventrally from L4 spiny stellate cells, mainly in the column, but with some overlap into neighboring columns, including subgranular layers (Lubke et al. 2000; Narayanan et al. 2015). Basal dendritic branches of L5 pyramidal cells spanning into the neighboring barrel could be engaged by axons confined to the home column. Glutamate uncaging studies in row-deprived mouse barrel cortex demonstrate that L4 connections from neighboring spared columns to L5IB in the deprived column can potentiate in response to deprivation (Jacob et al. 2012), but this has not been demonstrated for L5RS cells. Finally, L6 cells, known to display short-latency responses to whisker stimulation (Constantinople and Bruno 2013; de Kock et al. 2007) and to project axons to L5 (Narayanan et al. 2015), are alternative candidates for inducing the short-latency responses in L5RS cells. However, neither L4 nor L6 inputs to L5RS cells were found to potentiate (Jacob et al. 2012).

#### Pathways underlying spontaneous activity in L5.

Information about the principal whisker decreases significantly within the deprived column for all layers except L4, whereas information about the spared whiskers increases for all layers except L3. In addition, spared whisker information builds up in the L5RS cells after stimulation more rapidly than in control cases, partly due to potentiation of a short-latency excitatory input (vide supra) but also due to a decrease in spontaneous activity. Spontaneous activity varies according to the anesthetic level, the sleep states, or, in the awake animal, the conscious state, and it might alter the Chernoff information for the RS cells and other cell types. Urethane anesthesia creates a state of delta-wave activity where the EEG fluctuates at 0.5–4 Hz due to the burst-pause activity of L5 neurons, similar to the condition present in natural slow-wave sleep (Armstrong-James et al. 1985; Armstrong-James and Fox 1988). The burst activity is generated partly by intracortical circuits, particularly via horizontal connections in L5 (Beltramo et al. 2013; Chauvette et al. 2010; Le Bon-Jego and Yuste 2007), and partly by thalamic input originating in principal and intralaminar thalamic nuclei (David et al. 2013; Doi et al. 2007; Fox and Armstrong-James 1986). The decrease in spontaneous activity in the L5RS cells was accompanied by a hyperpolarization in membrane potential, suggesting a decrease in excitatory drive. The decrease in spontaneous activity would not appear to arise from a change in principal thalamic input, because this appears to potentiate in RS cells; it also would not appear to derive from a decrease activity in the L3-to-L5 pathway, because L5 bursts of action potentials persist when activity is abolished in supragranular layers (Beltramo et al. 2013), and some degree of independence was observed between subgranular and supragranular spontaneous activity (Reyes-Puerta et al. 2015). This suggests that a decrease in horizontal connections between L5 cells, which are known to be important for spontaneous bursts of action potentials (Beltramo et al. 2013; Chauvette et al. 2010), are more influential for the decrease in spontaneous activity. In this respect, it is of interest that bursts of action potentials in IB cells tend to precede those in RS cells (Chauvette et al. 2010), which suggests that IB-to-RS cell connections may be depressed by whisker deprivation. Conversely, the IB cells still show normal levels of spontaneous activity, which is consistent with the lack of depression of columnar pathways and potentiation of intracolumnar pathways onto L5IB cells (Jacob et al. 2012).

#### Compound sources of principal whisker depression.

**Fig. 8.**
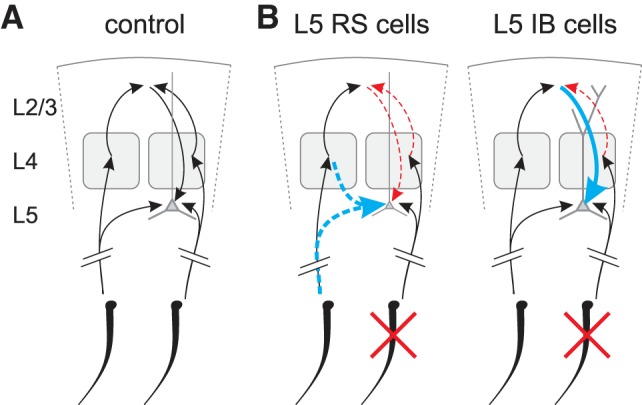
Schematic depiction of the proposed model of circuit plasticity for L5 neurons. *A*: control case. Sensory information from the whiskers (*bottom*) reaches L5 via two pathways, a direct thalamic input or through a cortical relay L4 → L2/3 → L5. Two adjacent cortical columns are represented, but for simplification a single L2/3 spot is drawn due to L2/3 relative homogeneity. The model can easily be implemented to distinguish the columns in L2/3. We do not distinguish L5 IB and RS cells for the control case. *B*: proposed circuit plasticity for L5RS cells (*left*) and L5IB cells (*right*). Depression of pathway strength is indicated with dashed red arrows, and potentiation with thick blue arrows. For RS cells, a potentiation upstream of L2/3 should be considered. The potentiation could be from direct thalamocortical synapses, but we cannot exclude a L4 → L5 potentiation, which is why the connection is included with a dashed blue arrow. For IB cells, potentiation arises at L2/3 → L5 synapses.

L2/3 provides a major input to L5 across various cortical areas (Hooks et al. 2011). In the barrel cortex, input from L2/3 cells to L5b is important for principal whisker responses in L5b (Wright and Fox 2010). In the current row-deprivation experiments, the input from L2/3 to L5 is depressed within the deprived column, and this is due to two main effects; first, the L4 input to L2/3 is depressed in the deprived column (Glazewski and Fox 1996; Glazewski et al. 1998), and second, the L2/3 inputs to L5 are depressed for RS but not IB cells (Jacob et al. 2012). This set of circumstances would be expected to create a depression in L5IB cells and a greater depression in L5RS cells; thus our observations support this model (Fig. 8). A recent deprivation study in mice showed a loss of dendritic spines in L4 pyramidal cells following whisker trimming (Miquelajauregui et al. 2015). L4 pyramidal and spiny stellate cells could not always be distinguished in the present study, so we did not analyze them separately. We did not find any significant changes in the L4 cells independent of subtype. A more specific depression of L4 inputs might impact L5 plasticity. One other factor that might contribute to the lower depression of L5IB cells is that the hyperpolarization activated current (*I*_h_) is downregulated in the apical dendrites by whisker deprivation, and this effect appears restricted to IB cells (Breton and Stuart 2009). Lower levels of *I*_h_ would be expected to increase excitability of the IB cells to their diminished L2/3 input and to some extent counter the circuit effects.

#### Excitatory vs. inhibitory influences in expression of plasticity.

Inhibition is important for induction of plasticity; disinhibition occurs phasically and chronically during the early stages of whisker deprivation in superficial layers (House et al. 2011; Katzel and Miesenbock 2014; Kelly et al. 1999; Li et al. 2014), but its role in expression of barrel cortex plasticity is less well established. For the spared whiskers, both excitatory and inhibitory conductances were found to increase in L5RS cells, although at different latencies, which rules out the idea of disinhibition and supports the idea that potentiation is due to excitatory inputs likely through excitatory synapse potentiation. For the spared whiskers, the increase in *g*_E_ preceded that in *g*_I_ and therefore allowed excitation to escape the influence of the inhibitory effect and depolarize the cell. For the deprived whiskers, the concomitant increase of *g*_E_ and *g*_I_ canceled one another out and led to no change. This finding is consistent with previous studies showing that the phase relationship between excitation and inhibition is crucial in creating the response selectivity of neurons in the barrel cortex (Wilent and Contreras 2005). Our results should be interpreted with caution, since all studies estimating conductance from somatic recordings are biased toward perisomatic synapses (Williams and Mitchell 2008). For L5 pyramidal cells, all excitatory input pathways target the perisomatic dendrites in L5, even if VPm neurons also contact oblique dendrites in L4 and L2/3 inputs also target the apical tuft (Petreanu and Svoboda 2009). For inhibitory inputs, parvalbumin-expressing interneurons target the perisomatic dendrites of L5 pyramidal cells, but somatostatin-expressing interneurons essentially target the apical tuft (reviewed in Naka and Adesnik 2016). The plasticity of inputs from the latter interneuron type cannot be assessed with somatic conductance estimation and need to be explored with alternative methods.

#### Conclusions.

The present analysis shows that when information content is taken into account, a relatively minor spared input potentiation in L5RS cells, when combined with a reduction in spontaneous activity, retrieves greater information content than potentiation of spared whisker responses in the presence of spontaneous activity for the L5IB cells. In conclusion then, the present data indicate that in addition to differences in underlying plasticity mechanisms induced in L5RS and IB cells due to their different propensities for Hebbian vs. homeostatic plasticity (Greenhill et al. 2015), cortical and subcortical circuit effects act to exacerbate the differences in plasticity in different cell types (Fig. 8). Despite different underlying mechanisms, L5RS and L5IB cells both act to increase the information content of spared whisker responses.

## GRANTS

We acknowledge funding from RIKEN Brain Science Institute (to A. Mitani and T. Toyoizumi), from the Brain/MINDS (Brain Mapping by Innovative Neurotechnologies for Disease Studies) supported by the Ministry of Education, Science, and Technology (MEXT) of Japan (to T. Toyoizumi), and from the Medical Research Council (to K. Fox) for this work.

## DISCLOSURES

No conflicts of interest, financial or otherwise, are declared by the authors.

## AUTHOR CONTRIBUTIONS

V.J. and K.F. conception and design of research; V.J. performed experiments; V.J., A.M., T.T., and K.F. analyzed data; V.J., A.M., T.T., and K.F. interpreted results of experiments; V.J., A.M., and T.T. prepared figures; V.J., T.T., and K.F. drafted manuscript; V.J., A.M., T.T., and K.F. edited and revised manuscript; V.J., A.M., T.T., and K.F. approved final version of manuscript.
